# The application and effectiveness of acceptance and commitment therapy on psychological resilience in the primary caregivers of ostomy patients: A randomized controlled trial

**DOI:** 10.1097/MD.0000000000050303

**Published:** 2026-08-28

**Authors:** Li Teng, Xin Wu, Huili Xu, Yun Wang, Tian Luo, Adan Fu

**Affiliations:** a Wound and Ostomy Clinic, The Central Hospital of Wuhan, Tongji Medical College, Huazhong University of Science and Technology, Wuhan, Hubei, China; b Department of General Surgery, The Central Hospital of Wuhan, Tongji Medical College, Huazhong University of Science and Technology, Wuhan, Hubei, China; c Department of Nursing, The Central Hospital of Wuhan, Tongji Medical College, Huazhong University of Science and Technology, Wuhan, Hubei, China.

**Keywords:** acceptance and commitment therapy, ostomy, primary caregivers, psychological resilience

## Abstract

**Background:**

This study aims to investigate the effects of acceptance and commitment therapy (ACT) on enhancing psychological resilience, psychological flexibility, caregiving capacity, and benefit-finding among caregivers of ostomy patients.

**Methods:**

A total of 106 primary caregivers of ostomy patients were recruited from a tertiary hospital in Wuhan. Participants were randomly assigned to the intervention or control group in a 1:1 ratio using a computer-generated random sequence. Allocation was concealed using sequentially numbered, opaque, sealed envelopes prepared by a researcher not involved in recruitment or intervention delivery. The control group received standard nursing care, while the intervention group was provided with ACT in addition to standard care over a period of 12 weeks. The study utilized the 10-item Connor-Davidson Resilience Scale, Acceptance and Action Questionnaire-II (AAQ-II), Family Caregiving Task Inventory (FCTI), and Benefit Finding Scale to assess the outcomes before and after the intervention in both groups.

**Results:**

Post-intervention analysis showed that the intervention group had significantly lower AAQ-II scores and FCTI scores compared with the control group (AAQ-II: 19.57 ± 7.78 vs 24.04 ± 8.39; FCTI: 7.75 ± 3.69 vs 12.57 ± 6.76; *P* < .05), indicating improved psychological flexibility and caregiving ability. In contrast, the intervention group demonstrated significantly higher psychological resilience and benefit-finding scores compared with the control group (Connor-Davidson Resilience Scale: 24.94 ± 7.69 vs 20.89 ± 6.66; Benefit Finding Scale: 86.58 ± 7.47 vs 78.75 ± 8.12; *P* < .05).

**Conclusion:**

ACT was associated with improvements in psychological resilience, psychological flexibility, caregiving ability, and benefit finding among primary caregivers of ostomy patients. These findings suggest that ACT may be a useful supportive intervention for improving caregivers’ psychological adaptation and caregiving capacity.

## 1. Introduction

Statistical data indicates that the incidence and mortality rates of colorectal cancer in our country rank among the highest globally and are continuing to rise.^[1]^ With the ongoing increase in cancer survival rates, cancer has progressively become a chronic condition necessitating family-based care. Cancer not only affects the physical and mental health of patients but also has a significant impact on the health of their caregivers. Ostomy surgery alters the normal bowel movements of patients, causing immense psychological stress. Family caregivers play a crucial role in the overall recovery of patients who have undergone permanent colostomy, as their caregiving abilities, mental state, and quality of life directly influence the patient’s rehabilitation process.^[2]^ Most studies focus on the mental health of the ostomy patients themselves, while family caregivers, who provide long-term assistance during the treatment and recovery period, receive attention from only 27% of researchers.^[3]^ These caregivers often face substantial psychological pressure and caregiving burdens. When dealing with the nursing needs of ostomy patients, they frequently feel helpless, anxious, and avoidant. These negative emotions can affect their caregiving abilities and quality of life. If their psychological well-being is not promptly restored, it will impact both the overall quality of life of the caregivers and the quality of care provided to the patients.

Acceptance and commitment therapy (ACT), as a psychological treatment method, has been used by domestic and international scholars for the treatment of chronic diseases, depression, and other conditions. It represents the third wave of cognitive-behavioral therapy.^[4]^ ACT advocates embracing pain and accepting the reality that “happiness is not the norm.”^[5]^ Through 6 processes – acceptance, cognitive defusion, being present in the moment, self-as-context, clarifying values, and committed action – it helps individuals accept unchangeable emotions and thoughts, guiding them to take actions aligned with their values, thereby enhancing psychological flexibility.^[6]^

Recent evidence has increasingly supported the application of ACT among caregivers of individuals with chronic health conditions. A systematic review and meta-analysis reported that ACT significantly improved psychological flexibility and reduced psychological distress among informal caregivers.^[7]^ In addition, ACT-based interventions have demonstrated beneficial effects on caregiver burden, emotional well-being, and coping ability among caregivers of patients with advanced cancer, dementia, and other chronic illnesses.^[7,8]^ Previous studies have also suggested that psychological flexibility is an important mechanism through which ACT promotes adaptation to long-term caregiving stress.^[9]^ These findings suggest that ACT may represent a promising psychological intervention for caregivers facing persistent caregiving demands. More specifically, a recent systematic review and meta-analysis focusing on caregivers of people with cancer reported that ACT may reduce anxiety, depression, stress symptoms, and experiential avoidance while improving psychological flexibility.^[10]^ However, evidence remains limited for caregivers of patients with ostomies, particularly in colorectal cancer contexts, where caregiving involves both cancer-related stress and stoma-specific care demands.

Current research predominantly focuses on ostomy patients themselves, with limited clinical practices addressing the needs of their primary caregivers. Family caregivers of ostomy patients constitute a particularly vulnerable population because they must not only provide routine daily care but also manage stoma-related complications, assist with ostomy care procedures, and cope with patients’ emotional distress and altered body image. Previous studies have shown that caregivers of ostomy patients frequently experience caregiving burden, uncertainty, social restrictions, and emotional distress while assisting patients in adapting to life with a stoma.^[11]^ These long-term caregiving responsibilities often result in substantial psychological burden, including anxiety, depression, emotional exhaustion, and caregiving strain. Because caregiving-related stressors are often persistent and difficult to eliminate, ACT may be particularly suitable for this population. Rather than focusing solely on symptom reduction, ACT aims to enhance psychological flexibility and help individuals adapt to ongoing challenges through acceptance, mindfulness, values clarification, and committed action. Therefore, this study sought to apply ACT as an intervention for primary caregivers of colorectal cancer patients with ostomies and to evaluate its effects on psychological resilience, psychological flexibility, caregiving ability, and benefit finding.

## 2. Methods

### 2.1. Study design and participants

This study was designed as a single-center, parallel-group randomized controlled trial with pre- and post-intervention assessments. The study was conducted among primary caregivers of patients who underwent ostomy surgery in the gastrointestinal surgery department of a tertiary hospital in Wuhan, Hubei Province, China, between May 2025 and October 2025. This study has been approved by the Ethics Committee of Wuhan Central Hospital (Approval Number: WHZXKYL2025-113). This trial was retrospectively registered in the Chinese Clinical Trial Registry (ChiCTR2500108230) on August 27, 2025. All participants provided written informed consent before being enrolled in the study. Eligible participants were randomly assigned to either the intervention group or the control group in a 1:1 ratio using a computer-generated random sequence. Allocation was concealed using sequentially numbered, opaque, sealed envelopes prepared by a researcher not involved in recruitment or intervention delivery. Participants were followed for 12 weeks after enrollment. No participants dropped out during the 12-week follow-up; therefore, all 106 participants were included in the final analysis. Due to the nature of the intervention, participants and intervention providers could not be blinded to group allocation. However, outcome assessments and data collection were conducted by independent assessors who were blinded to treatment allocation. The ACT intervention was delivered by trained personnel who were not involved in outcome assessment or data analysis.

The inclusion criteria were as follows: aged 18 years or older; met the diagnostic criteria for colorectal cancer^[12]^ and were the caregivers of ostomy patients who had undergone ostomy surgery in this hospital; primarily responsible for the care tasks of the ostomy patient during hospitalization, with a daily care duration of 4 hours or more; and caregivers voluntarily participated, consented, and signed an informed consent form. The exclusion criteria were: having received psychological intervention prior to enrollment; ineffective communication or cognitive dysfunction; and a history of diagnosed psychiatric disorders that could interfere with study participation or outcome assessment.

Sample size was calculated based on the primary outcome of psychological resilience using the formula for comparing two independent means: n = 2(*Z*α/2 + *Z*β)^2^σ^2^/δ^2^, where α was set at 0.05 (two-sided) and β at 0.10 (power = 90%). Based on a pilot study involving 30 caregivers conducted prior to the formal trial, the expected between-group difference (δ) was estimated to be 3.27 and the standard deviation (σ) was estimated to be 4.88. The required sample size was calculated as 48 participants per group. Considering a potential dropout rate of 10%, the final sample size was increased to 53 participants per group, resulting in a total sample size of 106 participants.

### 2.2. Intervention method

In the control group, standard nursing practices were administered, encompassing postoperative care instructions, dietary guidance, procedures for changing the ostomy bag, and the observation and management of ostomy-related complications. Routine nursing care was initiated for both groups on the first postoperative day and continued until discharge. After discharge, both groups received weekly follow-up for 12 weeks. In addition, the intervention group received the ACT intervention during the same 12-week period.

The intervention group received ACT in addition to the routine care provided to the control group. This intervention included the following key components: first, formation of a Professional Care Team: This team comprised attending physicians, head nurses, certified ostomy care specialists, and other nursing staff. All team members underwent ACT training organized by a professional psychotherapist, emphasizing critical aspects and challenges in the daily care of ostomy patients. Second, intervention program design: based on the 6 steps of the ACT model, namely acceptance, cognitive defusion, being present, self-as-context, values clarification, and committed action, and considering the promoting and hindering factors of psychological health in caregivers of patients with colostomy, an ACT intervention program for caregivers of patients with colostomy was designed and formulated. During the hospital stay, the intervention was conducted within the hospital ward. After discharge, interventions were conducted through outpatient visits, Tencent Meetings, or phone calls. A reminder telephone call was made one day before each session. The intervention was conducted either face-to-face or individually once per week. The ACT intervention is implemented over 12 weeks, totaling 6 sessions, with each session lasting 45 to 60 minutes. The specific intervention contents are shown in Table 1.

**Table 1:** Intervention content of acceptance and commitment therapy of primary caregivers of ostomy patients.

Themes	Intervention goals	Intervention content
Acceptance	Facilitate acceptance of enterostomy reality. Aid caregivers in acknowledging the existence of enterostomy, and developing the ability to observe and accept adverse emotions.	Comprehend ACT and foster communication and introduce the principles of Acceptance and Commitment Therapy (ACT), establish effective communication, secure consent and cooperation, and motivate caregivers to actively confront and understand enterostomy without avoidance, thereby alleviating resistance and tension when facing colostomy. Inform them that enterostomy is a therapeutic intervention aimed at disease management and health restoration. Encourage primary caregivers to view the experience of caring for patients as a life-enriching journey, engaging with it wholeheartedly. Promote the expression of inner feelings and thoughts, assisting caregivers in recognizing these emotions as normal responses, avoiding excessive expression or restraint. Guide caregivers to attend to and describe various emotions encountered during the care process, such as anxiety, anger, and helplessness. For instance, encourage caregivers to allocate 10 to 15 min daily to record emotional diaries, noting negative emotions and triggering events. Equip caregivers with relaxation techniques, including progressive muscle relaxation and abdominal breathing exercises.
Cognitive defusion	Cognitive dissociation education and teach the concept of cognitive dissociation, enabling caregivers to observe their own negative emotions and guide them in detaching from erroneous cognitions to foster a correct perception of the stoma.	Equip caregivers with the concept of cognitive dissociation, allowing them to reevaluate negative emotions such as feelings of inferiority and shame associated with caring for stoma patients from a third-person perspective, and address concerns about inadequate patient care due to stoma management challenges. Through situational case analysis, engage caregivers in discussions about misconceptions encountered during caregiving, helping them fully realize that these are subjective interpretations, objective realities. Facilitate the recognition of irrationality of these cognitions through questioning and exemplification, organize joint discussions, share coping strategies, and guide the formation of a more positive and rational cognitive framework.
Experiencing the present	Enhance present-moment awareness. Utilize mindfulness practices to improve caregivers, present-moment consciousness, release inherent obsessions, and focus on the benefits brought by the stoma.	Encourage present-moment attention: Motivate caregivers to consciously attend to their current situations and feelings, informing them of the importance of actively facing diseases, engaging with the present to shape a better future, and experiencing the support of relatives and medical staff. Through group discussions and case analyses, explore “skills acquired and social support felt during the caregiving period for ostomy patients,” guiding caregivers to ponder, reflect on, and clarify their values in the care process, and the guiding significance of these values to their caregiving behaviors. Establish an enterostomy WeChat communication group and official account to disseminate information about enterostomy, encouraging primary caregivers to actively communicate and share experiences within the group for emotional support.
Self-as-context awareness	Learn the concept of self-as-context. Introduce the concept of taking oneself as the scene, detect unreasonable self-cognitions, and assist caregivers in observing and examining themselves from a self-awareness perspective.	Instruct caregivers to focus their self-awareness purposefully, helping them distinguish between the conceptualized self and the true self, enabling them to swiftly escape erroneous cognitions. Encourage objective observation of real experiences in the current situation, accepting that “patients are integral family members, no different from others, and there is no shame in caring for ostomy patients.” Promote active cooperation with medical staff in patient care, and assist caregivers in learning methods, procedures, and steps for stoma replacement, as well as observation and treatment methods for stoma-related complications, by distributing flow charts and management manuals, thereby stimulating the primary role of caregivers and enhancing their nursing capabilities.
Clarifying values	Self-understanding through Behavioral Training. Engage in behavioral training to gain a profound understanding of oneself, clarify values, and set goals for value realization.	Clarify values in ACT: The values in ACT pertain to the lifestyle that primary caregivers aspire to and select. Team members present the patient’s diagnosis and treatment plan to primary caregivers, informing them of advancements in modern medical technology, guiding them to recognize their personal value orientations, assisting in establishing correct values, and encouraging a positive and optimistic approach to life, rediscovering their own values and life significance. Guide caregivers to contemplate and clarify core values in the care process, such as family responsibility and patient care, and the guiding significance of these values to their caregiving behaviors. Encourage the formulation of specific care plans and action plans according to personal values, actively practicing these values in daily care, such as committing to daily mindfulness training.
Committed action	Implement action plans and assist caregivers in breaking the action plan and applying it to the current care process.	Formulate care plans and programs based on individual conditions, establish incentive mechanisms, commend and reward primary caregivers who achieve their goals, continuously encourage caregivers, and instill confidence and a sense of achievement in patient care. Organize professional nursing training courses to instruct caregivers in correct ostomy nursing methods, complications prevention and treatment skills, nutritional support knowledge, etc, according to the nursing needs of enterostomy patients, thereby enhancing their nursing abilities.

The ACT intervention was delivered by a registered psychotherapist. Prior to study implementation, the intervention personnel completed ACT-related institutional training and participated in professional ACT workshops. A standardized intervention manual and structured session content were used throughout the study to ensure consistency of intervention delivery, and all sessions followed the protocol outlined in Table 1. Caregivers who were unable to attend scheduled sessions were provided with recorded sessions for later review to facilitate continued access to the intervention content. Although formal intervention fidelity assessments and attendance rates were not systematically recorded, these procedures were implemented to enhance intervention standardization and continuity. This issue has been acknowledged as a limitation, and future studies should include formal fidelity assessment, therapist adherence rating, and complete attendance recording.

### 2.3. Instrumentation

#### 2.3.1. Psychological resilience

The 10-item Connor-Davidson Resilience Scale^[13]^ was used to assess psychological resilience before and after the intervention in both groups. The scale consists of 2 dimensions: strength and toughness, with a total of 10 items. A 5-point Likert scoring method is applied, with each item scored from 0 to 4, for a total possible score of 0 to 40. Higher scores indicate better psychological resilience, and the scale’s Cronbach α coefficient is 0.916.

### 2.4. Psychological flexibility

The Acceptance and Action Questionnaire-2nd Edition, translated by Cao Jing,^[14]^ was used to evaluate psychological flexibility. The scale contains 7 items, scored on a 1 (never) to 7 (always) point scale. The total score is the sum of the 7 items, with a maximum of 49 points. Higher scores indicate a lower degree of psychological flexibility, and the Cronbach α coefficient for the scale is 0.883.

### 2.5. Caregiving ability

The Family Caregiver Task Inventory (FCTI), revised by Sun Jing et al in 2018, was used to assess caregiving ability among ostomy caregivers in mainland China.^[15]^ The scale uses a 3-point Likert scoring: 0 (not difficult), 1 (difficult), and 2 (very difficult), with a total of 25 items. The total score ranges from 0 to 50, with higher scores indicating more difficulties faced by the caregiver in the care process and weaker caregiving ability. The Cronbach α coefficient for this scale is 0.873.

### 2.6. Benefit finding

The Benefit Finding Scale for Adults, translated and reported by Liu Zhun et al,^[16]^ and used by Bian Jing^[17]^ for cancer patient caregivers, includes 5 dimensions: acceptance, family relationships, personal growth, social relationships, and health behaviors, with a total of 22 items. Each item is scored on a 5-point Likert scale from “not at all” to “a very great deal” with scores from 1 to 5. The total score ranges from 22 to 110, with higher scores indicating a greater sense of benefit finding from the illness. The Cronbach α coefficient for this scale is 0.887.

### 2.7. Data analysis

The data were entered and verified independently by 2 researchers to ensure accuracy. Statistical analyses were performed using SPSS version 27.0. The normality of continuous variables was assessed prior to analysis.

Categorical variables were presented as frequencies and percentages, and comparisons between groups were performed using the chi-squared test. Continuous variables were expressed as mean ± standard deviation. Intergroup comparisons were conducted using independent samples *t*-tests. A two-tailed *P* value of < .05 was considered statistically significant.

To address potential baseline imbalances between groups, sensitivity analyses were performed using analysis of covariance. The models included group assignment as the independent variable, post-intervention outcomes as dependent variables, and corresponding baseline values, education level, and ostomy type as covariates.

## 3. Results

### 3.1. Baseline characteristics

No participants discontinued the intervention or were lost to follow-up, and all randomized participants were included in the final analysis. In this study, a total of 106 participants were included, with 53 participants in the intervention group and 53 in the control group. The comparison of baseline characteristics between the 2 groups showed no statistically significant differences (*P* > .05). In the control group, there were 15 males and 38 females, aged from 28 to 82 years, with an average age of (56.00 ± 11.68) years; 23 individuals had a junior high school education or below, 22 cases were ileostomies, 17 cases were permanent ostomies, and 10 cases had complete understanding of ostomy; in the intervention group, there were 18 males and 35 females, aged from 31 to 75 years, with an average age of (55.79 ± 8.26) years; 27 individuals had a junior high school education or below, 20 cases were ileostomies, 26 cases were permanent ostomies, and 17 cases had complete understanding of ostomy. For detailed information, refer to Table 2.

**Table 2:** Baseline characteristics of participants in the 2 groups.

Variable name	Control group (n = 53)	Intervention group (n = 53)	*t/χ^2^*	*P*
Age (mean ± SD)	56.00 ± 11.68	57.13 ± 10.00	−0.536	.593
Gender, n (%)				
Man	15 (28.3)	18 (34.0)	0.396	.529
Woman	38 (71.7)	35 (66.0)		
Place of residence, n (%)				
Town	39 (73.6)	33 (62.3)	1.559	.212
Rural area	14 (26.4)	20 (37.7)		
Marital status, n (%)				
Have a spouse	48 (90.6)	46 (86.8)	0.376	.540
Without spouse	5 (9.4)	7 (13.2)		
Education level, n (%)				
Primary school and below	6 (11.3)	14 (26.4)	4.461	.216
Junior middle school	17 (32.1)	13 (24.5)		
High school or technical secondary school	17 (32.1)	17 (32.1)		
College degree or above	13 (24.5)	9 (17.0)		
Monthly family income (RMB)				
< 5000	25 (47.2)	34 (64.2)	3.168	.205
5000–10,000	23 (43.4)	15 (28.3)		
≥10,000	5 (9.4)	4 (7.5)		
Occupational status, n (%)				
Unemployed	6 (11.3)	11 (20.8)	3.186	.203
Employed	22 (41.5)	25 (47.2)		
Retired	25 (47.2)	17 (32.0)		
Relations with patients, n (%)				
Spouse	28 (52.8)	33 (62.3)	1.216	.749
Sons and daughters	18 (34.0)	13 (24.5)		
Father and mother	1 (1.9)	1 (1.9)		
Other	6 (11.3)	6 (11.3)		
Self-rated health, n (%)				
Poor	2 (3.8)	4 (7.5)	1.329	.514
Fair	13 (24.5)	16 (30.2)		
Good	38 (71.7)	33 (62.3)		
The stoma classification, n (%)				
Ileum	22 (41.5)	20 (37.7)	0.158	.691
Colon	31 (58.5)	33 (62.3)		
Ostomy properties, n (%)				
Permanent stoma	17 (32.1)	26 (49.1)	3.169	.075
Temporary stoma	36 (67.9)	27 (50.9)		

SD = standard deviation.

### 3.2. Psychological resilience

Before the intervention, there were no significant differences in the psychological resilience scores and various comparisons between the 2 groups of ostomy caregivers (*P* > .05). After 12 weeks of intervention, the psychological resilience scores and all related scores of the primary caregivers in both groups increased, with the intervention group scoring higher than the control group (*P* < .05, Table 3).

**Table 3:** Comparison of psychological resilience scores between the 2 groups (mean ± SD).

Group	Power		Toughness		Total points	
	Before the intervention	After the intervention	Before the intervention	After the intervention	Before the intervention	After the intervention
Control group (n = 53)	8.77 ± 3.20	10.83 ± 3.51*	8.60 ± 3.14	10.81 ± 3.36*	17.38 ± 5.98	20.89 ± 6.66*
Intervention group (n = 53)	8.58 ± 3.35	12.60 ± 4.07*	9.53 ± 3.18	12.34 ± 3.79*	18.11 ± 6.08	24.94 ± 7.69*
*t*	0.297	−2.40 2	−1.507	−2.195	−0.628	−2.904
*P*	.767	.018	.135	.030	.531	.004

SD = standard deviation.

* *P* < .05 compared to preintervention.

### 3.3. Psychological flexibility

Before the intervention, there was no significant difference in the psychological flexibility scores between the 2 groups of ostomy caregivers (*P* > .05). After 12 weeks of intervention, the psychological flexibility scores decreased in both groups, indicating that the intervention group had a higher level of psychological flexibility compared to the control group after the intervention, with the intervention group scoring lower than the control group (*P* < .05, Table 4).

**Table 4:** Comparison of psychological flexibility scores between the 2 groups (mean ± SD).

Group	Total score of mental flexibility	
	Before the intervention	After the intervention
Control group (n = 53)	25.58 ± 7.15	24.04 ± 8.39*
Intervention group (n = 53)	24.79 ± 7.78	19.57 ± 7.78*
*t*	0.369	2.846
*P*	.713	.005

SD = standard deviation.

* *P* < .05 compared to preintervention.

### 3.4. Caregiver care capacity

Before the intervention, there was no difference in the scores of the 2 primary caregivers (*P* > .05). After 12 weeks, FCTI scores decreased in both groups. The intervention group had significantly lower FCTI scores than the control group, indicating fewer caregiving difficulties and better caregiving ability (*P* < .05, Table 5).

**Table 5:** Comparison of caregiver care capacity scores between the 2 groups (mean ± SD).

Group	Adapt to caring for roles		Strain and provide assistance		Dealing with personal emotional needs	
	Before the intervention	After the intervention	Before the intervention	After the intervention	Before the intervention	After the intervention
Control group (n = 53)	3.72 ± 1.98	2.89 ± 1.88*	2.91 ± 1.61	2.45 ± 1.81*	3.45 ± 1.96	2.92 ± 2.15*
Intervention group (n = 53)	3.83 ± 2.11	1.66 ± 1.36*	2.75 ± 1.63	1.75 ± 1.11*	3.32 ± 2.12	2.02 ± 1.39*
*t*	−0.285	3.999	0.480	2.391	0.333	2.576
*P*	.776	<.001	.632	.019	.740	.011

Group	Evaluate family and community resource needs		Adjust your personal and care needs		total points	
	Before the intervention	After the intervention	Before the intervention	After the intervention	Before the intervention	After the intervention
Control group (n = 53)	2.79 ± 1.91	1.98 ± 1.82*	2.81 ± 1.78	2.34 ± 1.65*	15.79 ± 7.10	12.57 ± 6.76*
Intervention group (n = 53)	2.70 ± 1.73	1.19 ± 1.32*	2.68 ± 1.77	1.11 ± 1.01*	15.36 ± 7.04	7.75 ± 3.69*
*t*	0.267	2.565	0.383	4.608	0.316	2.216
*P*	.790	.012	.702	<.001	.753	<.001

SD = standard deviation.

* *P* < .05 compared to preintervention.

### 3.5. Disease benefit

There was no difference in the disease benefit scores of the primary caregivers of ostomy patients (*P > *.05); after 12 weeks of intervention, benefit-finding scores increased in both groups, and the intervention group had significantly higher scores than the control group after the intervention (*P* < .05, Table 6).

**Table 6:** Comparison of disease benefit between the 2 groups ($\bar{X}\pm \;s$).

Group	Accept		Family relation		Personal growth	
	Before the intervention	After the intervention	Before the intervention	After the intervention	Before the intervention	After the intervention
Control group (n = 53)	8.08 ± 2.56	9.09 ± 2.87*	21.58 ± 2.14	23.09 ± 2.40*	22.85 ± 3.77	24.42 ± 3.91*
Intervention group (n = 53)	8.75 ± 2.66	11.15 ± 2.33*	21.70 ± 2.28	24.28 ± 3.07*	23.42 ± 3.86	26.83 ± 4.11*
*t*	−1.344	−4.048	−0.263	−2.221	−0.763	−3.102
*P*	.182	<.001	.793	.029	.447	.002

Group	Social relationships		Health behavior		Total points	
	Before the intervention	After the intervention	Before the intervention	After the intervention	Before the intervention	After the intervention
Control group (n = 53)	10.49 ± 2.13	10.79 ± 2.16*	11.11 ± 2.49	11.32 ± 2.86*	74.15 ± 7.92	78.75 ± 8.12*
Intervention group (n = 53)	10.30 ± 2.16	11.66 ± 1.59*	10.92 ± 2.52	12.66 ± 1.72*	75.02 ± 7.77	86.58 ± 7.47*
*t*	0.453	−2.354	0.388	−3.41	−0.569	−5.164
*P*	.652	.020	.699	.001	.570	<.001

* *P* < .05 compared to preintervention.

### 3.6. Adjusted analysis

Sensitivity analyses using analysis of covariance showed that the between-group differences in the primary outcomes remained statistically significant after adjustment for baseline values, education level, and ostomy type (all *P* < .05). These findings were generally consistent with the unadjusted analyses.

## 4. Discussion

### 4.1. Effects of ACT on psychological flexibility and resilience

Patients who have undergone colostomy and their caregivers often face significant psychological stress. Caregivers may experience negative emotions such as anxiety and depression due to the emotional challenges of the patient.^[18]^ ACT employs acceptance strategies to help caregivers learn to recognize and accept the presence of these emotions, rather than attempting to suppress or eliminate them, thereby alleviating psychological burden.^[19]^ Additionally, ACT emphasizes psychological flexibility, enabling caregivers to adapt their cognitive and behavioral responses when faced with the patient’s emotional fluctuations and caregiving demands. Through this intervention, caregivers can better manage their own emotions and reduce the psychological fatigue associated with long-term caregiving.^[9]^ Research findings demonstrate that following ACT intervention, caregivers in the intervention group exhibited significantly higher levels of psychological resilience and flexibility compared to those in the control group, aligning with prior research.^[20,21]^ These findings are also consistent with the systematic review by Ye et al, which demonstrated that ACT-based interventions improve psychological flexibility and reduce psychological distress among informal caregivers of individuals with chronic health conditions.^[7]^ A diagnosis of colorectal cancer represents a significant stressor for both patients and their families. Ostomy patients, in particular, face the dual challenges of cancer and ostomy, which can lead to heightened anxiety, depression, and other emotional responses, often resulting in avoidant behaviors.^[22]^ The findings of this study suggest that ACT strategies may enhance individuals’ adaptive capacities when confronted with stress and challenges, thereby reducing caregivers’ tendencies toward avoidant behaviors and cognitive fusion. Consequently, this enhances their psychological flexibility and ability to manage the stress and uncertainty associated with caring for ostomy patients.

### 4.2. Comparison with previous ACT caregiver studies

The findings of the present study are generally consistent with previous ACT research conducted among caregiver populations. Ye et al reported that ACT interventions significantly improved psychological flexibility and emotional well-being among informal caregivers of individuals with chronic health conditions.^[7]^ Similarly, Montaner et al found that ACT effectively reduced burnout and improved psychological outcomes among professional dementia caregivers.^[8]^ Losada et al also compared cognitive-behavioral therapy and ACT in dementia family caregivers with depressive symptoms, providing additional evidence for ACT-based approaches in caregiver populations.^[23]^ In oncology settings, Mosher et al demonstrated that ACT-based interventions improved psychological adaptation among patients with metastatic cancer and their family caregivers.^[24]^ Collectively, these findings support the applicability of ACT across diverse caregiving contexts, including caregivers of patients with colorectal cancer undergoing ostomy surgery. In addition, pilot randomized and secondary analyses in advanced gastrointestinal cancer patient-caregiver dyads found that ACT may reduce caregiver burden and improve physical and psychological symptom outcomes.^[25,26]^ Compared with these studies, the present trial extends existing evidence by focusing specifically on primary caregivers of ostomy patients and by simultaneously evaluating psychological flexibility, resilience, caregiving ability, and benefit finding.

### 4.3. Potential mechanism of change

Psychological flexibility is widely regarded as the central mechanism underlying ACT. Psychological flexibility has also been described as a fundamental aspect of health and adaptive functioning.^[27]^ In the present study, the reduction in Acceptance and Action Questionnaire-II scores suggests improved psychological flexibility among caregivers receiving ACT. Enhanced psychological flexibility may have enabled caregivers to respond more adaptively to caregiving-related stress, thereby contributing to improvements in psychological resilience and benefit finding. Although formal mediation analyses were beyond the scope of the current study, future research should examine whether psychological flexibility mediates the relationship between ACT and caregiver outcomes.

### 4.4. Caregiving ability and role adaptation

The specific disease characteristics associated with ostomy necessitate that patients, who are often unable to care for themselves physically and may exhibit psychological rejection, require prolonged care from caregivers. Caregivers are tasked with the irregular cleaning of effluent and maintaining stoma hygiene, as well as assisting with the replacement of ostomy bags. At the same time, caregiving ability has a direct impact on the patient’s recovery process and their adaptation to social life.^[28]^ The primary caregiver serves as the emotional pillar and mainstay for the patient. In order to assist the patient in adapting to a new life, the family role and social function of the primary caregiver undergo significant changes, which invisibly increases their caregiving pressure.

ACT helps primary caregivers adapt to their caregiving role, enhances their psychological adaptability, and assists them in addressing issues encountered while caring for ostomy patients, thereby reducing their caregiving burden.^[7]^ After the intervention, caregiving ability improved in both groups, with significantly greater improvement observed in the ACT group. Good caregiving ability can reduce the incidence of complications in ostomy patients, improve prognostic outcomes, and enhance the patients’ quality of life.^[8]^ This may be attributed to the fact that, over time, primary caregivers in the control group gradually accepted the ostomy patients psychologically, became more proficient in the nursing process, or sought additional emotional support, thus reducing their caregiving burden.^[29]^ Caregivers often feel neglected and undervalued, and report being excluded from the patient’s care plan. It is crucial that caregivers are directly involved in the caregiving process, and that their opinions are taken seriously and incorporated into clinical decisions. Therefore, this suggests that medical staff should pay more attention to different caregivers, adopt individualized psychological interventions, help caregivers understand and accept ostomy, correctly face changes in body image, and provide more training in ostomy care skills.

### 4.5. Benefit finding and positive adaptation

Benefit finding, also referred to as perceived benefits, encompasses the positive cognitive and behavioral transformations that an individual discerns following illness or adversity.^[16]^ Previous studies have demonstrated that benefit finding is associated with better psychological adjustment, lower emotional distress, and improved quality of life among both patients with cancer and their family caregivers.^[30,31]^ Empirical research has further shown that caregivers of cancer patients generally report a moderate level of benefit finding, which is inversely associated with negative emotional experiences.^[32]^ Song et al suggested that higher levels of benefit finding may alleviate caregiving burden and promote more positive caregiving experiences.^[33]^

In this study, the sense of benefit from illness and related scores among primary caregivers of ostomy patients increased following the intervention. Notably, the intervention group, which underwent ACT, demonstrated significantly higher levels of benefit finding and its subdimensions compared to the control group. However, the overall level of benefit finding post-ACT intervention remained moderate or below, indicating room for further improvement. Caring for a cancer patient is often a significant challenge for caregivers. The ACT intervention strategy enabled caregivers to confront reality, embrace challenges, adapt to their caregiving role, and take on caregiving responsibilities. By clarifying their values, caregivers gained a greater sense of purpose and meaning during the caregiving process, while also accumulating experiential wisdom. Additionally, the experience of having a family member with cancer heightened caregivers’ awareness of the importance of health, prompting them to reflect on and modify previous unhealthy lifestyle habits.^[34]^ These findings suggest that ACT may help caregivers derive positive meaning from their caregiving experiences, thereby enhancing their psychological well-being.

## 5. Limitations

Several limitations should be acknowledged. First, this study was conducted at a single center, which may limit the generalizability of the findings. Second, the follow-up period was limited to 12 weeks; therefore, the long-term effectiveness of ACT could not be evaluated. Third, because of the nature of the intervention, participant blinding was not feasible, which may have introduced performance bias. Fourth, although randomization was used, some baseline characteristics, such as ostomy permanence, showed numerical imbalance. Moreover, cancer stage and detailed disease severity were not fully included in the analysis. Therefore, residual confounding may remain and should be considered when interpreting the intervention effects. Finally, although efforts were made to minimize communication regarding intervention content, both groups were recruited from the same department, and potential contamination between groups cannot be completely excluded. In addition, formal intervention fidelity assessment and complete attendance recording were not systematically conducted, which may limit the evaluation of intervention consistency.

Future multicenter studies with larger sample sizes and longer follow-up periods are warranted to further examine the long-term effects of ACT among caregivers of ostomy patients.

## 6. Conclusion

In summary, ACT was associated with improvements in psychological flexibility, psychological resilience, caregiving ability, and benefit finding among primary caregivers of ostomy patients. These findings suggest that ACT may be a useful supportive intervention for improving caregivers’ psychological adaptation and caregiving capacity. However, because this was a single-center study with a short follow-up period, the long-term effectiveness and generalizability of the findings require confirmation in larger multicenter randomized controlled trials.

## Acknowledgments

The authors thank all caregivers and patients who participated in this study.

## Author contributions

**Conceptualization:** Li Teng.

**Data curation:** Xin Wu, Yun Wang, Tian Luo.

**Formal analysis:** Xin Wu, Tian Luo.

**Investigation:** Xin Wu, Huili Xu, Yun Wang.

**Methodology:** Xin Wu, Huili Xu, Tian Luo.

**Project administration:** Adan Fu.

**Resources:** Li Teng, Huili Xu.

**Software:** Huili Xu, Yun Wang, Tian Luo.

**Supervision:** Adan Fu.

**Validation:** Huili Xu, Yun Wang, Adan Fu.

**Writing – original draft:** Li Teng, Xin Wu.

**Writing – review & editing:** Li Teng, Adan Fu.
